# New Drugs, Appliances, and Things Medical

**Published:** 1889-12-21

**Authors:** 


### NEW DRUGS, APPLIANCES AND THINGS MEDICAL.

[All preparations, appliances, novelties, etc., of which a notice is desired, should be sent to The Editor, The Lodge, Porchester Square, W.]

### THE DERBYSHIRE RESPIRATOR.

We have received from Miss Dranfield, Corporation Oaks, Nottingham, a specimen of the above. In shape, it re sembles the ordinary form of respirator, but it is lighter. It consists of a double, closely-worked layer of knitted woollen material, the outer side being black, the inner of a light pink colour. It fits over the mouth easily and is most comfortable, being retained in position by two light loops of elastic material passing over the ears.

In choosing a respirator, one has to consider whether

(a). It should cover the mouth alone ; or,

(b). Both mouth and nose.

In a large number of cases the former variety is all that is needed.

Having determined this point, the others to be considered are lightness, neatness, and, chief of all, capability of being cleaned. The "Derbyshire" can be recommended in all three of these requirements.

We have found that by putting a layer of Gamgee tissue inside, next to the mouth, the efficiency of the invention is increased. A little eucalyptus oil or other volatile antiseptic or balsamic agent can, in this way, be easily used. Dropped on the tissue, on the side next to the respirator, it medicates the air, both inspired and expired, without coming into actual contact with the skin or lips. In phthisical cases, particularly, it is most important that all articles contaminated by the breath should be thoroughly disinfected. Respirators coming under this heading, the "Derbyshire" answers the test, being made of washable materials. In the use of respirators there are several points of interest :

1. Always put the respirator on in a warm atmosphere ; never wait till in the hall, on the staircase, or even on the steps, in the open air, as is often done.

2. Keep several respirators, so that the one in use may be clean. There is as much need to clean a respirator as to wash a handkerchief.

3. Never put on another person's respirator. This is done by children in play, or by adults, to see how the appliance feels or looks. A more potent way of acquiring consumption, if the owner is phthisical, could not exist, apart from the natural distaste that one should have for such experiments. In the same way, it is as well to be careful in the borrowing of woollen wraps or scarves, which are often used by their owners as extemporised respirators.

